# Psychosocial Assessment of Candidates for Transplantation (PACT) Score Identifies High Risk Patients in Pediatric Renal Transplantation

**DOI:** 10.3389/fped.2019.00102

**Published:** 2019-03-26

**Authors:** Kyle W. Freischlag, Vivian Chen, Shashi K. Nagaraj, Annabelle N. Chua, Dongfeng Chen, Delbert R. Wigfall, John W. Foreman, Rasheed Gbadegesin, Deepak Vikraman, Eileen T. Chambers

**Affiliations:** ^1^School of Medicine, Duke University, Durham, NC, United States; ^2^Department of Pediatrics, Duke University, Durham, NC, United States; ^3^Department of Pathology, Duke University, Durham, NC, United States; ^4^Department of Surgery, Duke University, Durham, NC, United States

**Keywords:** renal transplantation, pediatrics, kidney, adherence, psychosocial factors

## Abstract

**Background:** Currently, there is no standardized approach for determining psychosocial readiness in pediatric transplantation. We examined the utility of the Psychosocial Assessment of Candidates for Transplantation (PACT) to identify pediatric kidney transplant recipients at risk for adverse clinical outcomes.

**Methods:** Kidney transplant patients <21-years-old transplanted at Duke University Medical Center between 2005 and 2015 underwent psychosocial assessment by a social worker with either PACT or unstructured interview, which were used to determine transplant candidacy. PACT assessed candidates on a scale of 0 (poor candidate) to 4 (excellent candidate) in areas of social support, psychological health, lifestyle factors, and understanding. Demographics and clinical outcomes were analyzed by presence or absence of PACT and further characterized by high (≥3) and low (≤2) scores.

**Results:** Of 54 pediatric patients, 25 (46.3%) patients underwent pre-transplant evaluation utilizing PACT, while 29 (53.7%) were not evaluated with PACT. Patients assessed with PACT had a significantly lower percentage of acute rejection (16.0 vs. 55.2%, *p* = 0.007). After adjusting for HLA mismatch, a pre-transplant PACT score was persistently associated with lower odds of acute rejection (Odds Ratio 0.119, 95% Confidence Interval 0.027–0.52, *p* = 0.005). In PACT subsection analysis, the lack of family availability (OR 0.08, 95% CI 0.01–0.97, *p* = 0.047) and risk for psychopathology (OR 0.34, 95% CI 0.13–0.87, *p* = 0.025) were associated with a low PACT score and post-transplant non-adherence.

**Conclusions:** Our study highlights the importance of standardized psychosocial assessments and the potential use of PACT in risk stratifying pre-transplant candidates.

## Introduction

Renal transplantation is the treatment of choice for end-stage renal disease in children and adolescents. Identification of psychosocial factors that can negatively impact post-transplant care is important to ensure successful clinical outcomes, which include adherence to medications, freedom from rejection and long-term patient and allograft survival. For children with end-stage renal disease, psychosocial stressors associated with increased morbidity and mortality include lower socioeconomic status and limited parental support (1, 2). Currently, there is no standardized approach to determine psychosocial readiness in pediatric kidney transplantation.

Multiple pre-transplant assessment tools have been developed for adults with scant data in pediatrics. These include the Stanford Integrated Psychosocial Assessment for Transplant (SIPAT), the Structured Interview for Renal Transplantation (SIRT), and the Transplant Evaluation Rating Scale (TERS) (3–5). The Pediatric Transplant Rating Instrument (P-TRI) attempted to address this gap in pediatric transplantation; however, follow-up studies have shown inconsistency with poor inter-rater reliability (6, 7). Other studies have simply used clinical judgement without a tool to risk-stratify patients (8).

The most widely used tool, the Psychosocial Assessment of Candidates for Transplantation (PACT), was developed in the 1980s as a standardized pre-transplant psychosocial evaluation tool (9). Since its development, PACT has shown good inter-rater reliability, improved clinical ease of use, and is a uniform framework for pre-transplant evaluation across all organ systems (9–11). As PACT has shown clinical utility and reliability in adult solid organ transplantation (12–15), we hypothesized that PACT could identify high risk pediatric transplant recipients and potentially improve transplant outcomes. In this pilot study, we aimed to assess the effect of implementing a standardized assessment such as PACT on clinical outcomes.

## Materials and Methods

### Study Design

In this retrospective analysis, eligible patients for inclusion were <21 years of age at time of transplantation, had continued post-transplant follow-up, and underwent renal transplantation for end-stage renal disease from January 1, 2005 to December 31, 2015 at Duke University Medical Center. Patients were excluded for missing transplant data or not receiving their primary transplantation or follow-up at our institution. The Duke University Institutional Review Board (IRB Protocol #0078991) approved this study.

### Study Outcomes

The primary endpoint of our study was biopsy proven acute rejection graded by Banff criteria within 3 years post-transplantation (16). Secondary endpoints included post-transplant dialysis, length of hospital stay, 30-day readmission, missed post-transplant appointments per year, non-adherence, renal allograft survival, and patient death. Non-adherence was determined by patient self-report, undetectable drug levels, or missed appointments (17, 18).

### Pact Score and Psychosocial Analysis

The PACT assessed candidates on 4 domains including social support, psychological health, lifestyle factors, and understanding of transplant and follow-up. PACT contained 8 subsection items: family support, family availability, personality factors, risk for psychopathology, ability to sustain change, drug and alcohol abuse, medical adherence, and relevant knowledge. The social support domain included evaluation of family support and family availability with an emphasis on relationships. The psychological health domain incorporated assessment of personality factors/psychiatric disorders and risk for psychopathology, which focused on coping mechanisms and family history of psychiatric disorders. The lifestyle factors domain evaluated dietary/exercise habits and the ability to change unhealthy behaviors, drug and alcohol use, and compliance with medications/medical advice. Finally, the understanding of transplant and follow-up domain encompassed relevant transplant knowledge and receptiveness to education. (19). Each category was assessed by scale of mild, moderate, or severe (9, 20). An overall final transplant candidacy score from 0 (contraindication to transplant) to 4 (excellent candidate) was assigned by the social worker. A low PACT score was defined as a score ≤ 2 and a high PACT score was defined as ≥3 (21). The psychosocial assessment was incorporated into the decision to transplant and patients with low PACT score were monitored more closely after transplant.

The psychosocial assessment was one of the initial steps for assessing transplant candidacy and was completed 1–2 months prior to listing for transplant. Trained licensed hospital social workers who specialize in pre-transplant evaluations performed our psychosocial assessments using language suitable for a 5th grade reading level. All psychosocial assessments were incorporated into multidisciplinary meetings to help evaluate the readiness of potential candidates alongside other concerns such as medical comorbidities. Preceding the PACT, all patients received a pre-transplant psychosocial analysis by a licensed social worker from 2005 to 2010. However, the pre-transplant psychosocial rating before the implementation of the PACT was not standardized, subject to variability in content (home life, finances, transportation, school, and adherence) depending on the evaluator and differed in the criteria for candidate suitability. The PACT was implemented to standardize pediatric renal pre-transplant assessments in 2010. All pre-transplants assessments were completed using PACT from 2010 to 2015. Final subsection scores represented the abilities and risk factors present in the primary caregivers and the child, starting at the developmental age of 12. Patients were listed regardless of PACT score. Increased pre-transplant visits with social worker intervention were initiated for patients with a low PACT score and transplanted only for score of >1. Patients with psychiatric illnesses were referred to outpatient psychiatry for titration of medications and counseling pre-transplant and not transplanted until issues controlled. Patients with low PACT scores defined as <2, were monitored more closely post-transplantation by medical team, social workers, and when appropriate by psychiatrist and/or psychologists. In cases with multiple psychosocial assessments over time, the PACT score closest to the time of transplant was used.

### Statistical Analysis

Patients were stratified based on presence or absence of a PACT assessment. Of those with a PACT assessment, sub-analysis by low vs. high PACT score was performed. Baseline characteristics and unadjusted outcomes were compared using the Kruskal-Wallis test for continuous variables and Pearson χ^2^ test for categorical variables. For acute rejection, a multivariable linear model was used adjusting for HLA mismatch. A model examining probability of freedom from acute rejection was generated using the Kaplan-Meier method.

A *p*-value of less than 0.05 was deemed statistically significant. Statistical analysis was performed using R version 3.3.1 (R Foundation for Statistical Computing, Vienna, Austria).

## Results

### Demographics

A total of 54 pediatric patients who underwent renal transplantation at Duke University from 2005 to 2015 met the inclusion criteria. Of these, 25 (46.3%) patients had a PACT assessment while 29 (53.7%) patients had no PACT assessment. Demographics and clinical characteristics between the two groups are shown in Table 1. Recipients with a PACT assessment were better matched (HLA mismatch 1.00, 2.52 *p* = 0.001), received a lower percentage of their allografts from living donors (24 vs. 55.2%, *p* = 0.04), and had less follow-up time (2.87 vs. 8.34 years, *p* < 0.001) when compared to their no PACT assessment counter parts. In PACT assessment recipients, the median PACT score was 3 (IQR 3.00–4.00). Additionally, 4 patients met the definition of a low PACT score (≤2) while 21 patients had a high PACT score (≥3) (21). Otherwise, there were no significant differences in gender, race, BMI, PRA, pre-transplant dialysis, PRA, immunosuppression regimen, viral serology status, diagnosis, re-transplant, or cold ischemia time between the two groups (Table 1).

**Table 1:** Demographic characteristics between recipients with and without a PACT assessment.

	**PACT assessment**	**No PACT assessment**	***p***
n	25	29	
Age at transplant (median [IQR])	14.80 [8.51, 17.08]	13.39 [4.12, 15.30]	0.249
Female (%)	9 (36.0)	8 (27.6)	0.711
**Race (%)**			0.766
African American	10 (40.0)	11 (37.9)	
Caucasian	10 (40.0)	14 (48.3)	
Other	5 (20.0)	4 (13.8)	
BMI at transplant (median [IQR])	22.00 [12.00, 35.00]	25.00 [12.00, 37.00]	0.952
HLA Mismatch [mean (sd)]	1.00 (1.55)	2.52 (1.70)	0.001
Class I PRA > 20% (%)	1 (4.0)	1 (3.4)	0.999
Class II PRA > 20% (%)	2 (8.0)	4 (13.8)	0.809
Pre-transplant dialysis (%)	19 (76.0)	17 (58.6)	0.289
**Induction (%)**			0.443
Anti-thymocyte Globulin	8 (33.3)	6 (21.4)	
IL-2 inhibitor	11 (45.8)	18 (64.3)	
None	4 (16.7)	2 (7.1)	
Other	1 (4.2)	2 (7.1)	
**MAINTENANCE IMMUNOSUPPRESSION**			
Calcineurin inhibitors	25 (100.0)	26 (89.7)	0.29
Steroids	25 (100.0)	26 (89.7)	0.29
Anti-metabolites	25 (100.0)	26 (89.7)	0.809
EBV immune	11 (44.0)	11 (37.9)	0.861
CMV immune	7 (28.0)	10 (34.5)	0.828
**Diagnosis (%)**			0.449
Dysplasia	5 (20.0)	12 (41.4)	
FSGS	8 (32.0)	8 (27.6)	
Glomerulonephritis	3 (12.0)	1 (3.4)	
Obstructive Uropathy	3 (12.0)	3 (10.3)	
Other	6 (24.0)	5 (17.2)	
Re-transplant (%)	1 (4.0)	2 (6.9)	0.999
Living donor (%)	6 (24.0)	16 (55.2)	0.041
Total ischemia (hours, median [IQR])	11.75 [2.18, 15.23]	1.50 [1.00, 16.00]	0.339
Follow-up time [years, mean (sd)]	2.87 (2.30)	8.34 (4.12)	<0.001

### Clinical Outcomes

**Figure 1:**
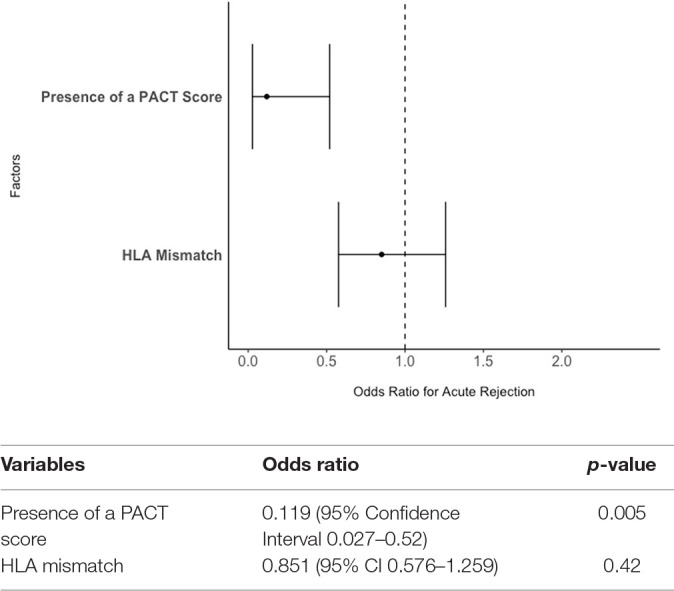
The effect of PACT score and HLA mismatch on the odds of acute rejection in pediatric kidney transplantation. The presence of a PACT score remained significantly associated with lower odds of rejection within 3-years post-transplant even after adjusting for HLA mismatch.

Unadjusted clinical outcomes between PACT assessment and no PACT assessment patients are displayed in Table 2. Patients with PACT assessment had a significantly lower percentage of acute rejection within the first 3 years post-transplant (16.0 vs. 55.2%, *p* = 0.007) and a lower percentage of non-adherence (28.0 vs. 55.2%, *p* = 0.08). After adjustment for HLA mismatch, lower odds of acute rejection remained associated with having a PACT score (Odds Ratio 0.119, 95% Confidence Interval 0.027–0.52, *p* = 0.005) but not HLA mismatch (OR 0.851, 95% CI 0.576–1.259) (Figure 1).

**Table 2:** Clinical outcomes in recipients with a PACT assessment and with no PACT assessment.

	**PACT assessment**	**No PACT assessment**	***p***
n	25	29	
Post-transplant dialysis (%)	9 (36.0)	12 (42.9)	0.819
Patient death [mean (sd)]	1.00 (0.00)	1.07 (0.26)	0.188
Missed appointments per year [mean (sd)]	0.44 (0.78)	0.17 (0.30)	0.108
Non-adherence (%)	7 (28.0)	16 (55.2)	0.082
Graft failure (%)	0 (0.0)	3 (10.3)	0.29
30-day readmission (%)	5 (20.0)	3 (10.3)	0.541
Hospital stay (days, median [IQR])	7.00 [5.00, 9.50]	7.00 [6.00, 10.00]	0.506
Acute rejection [mean (sd)]	4 (16.0)	16 (55.2)	0.007
Delayed graft function (%)	6 (25.0)	3 (10.3)	0.295

**Figure 2:**
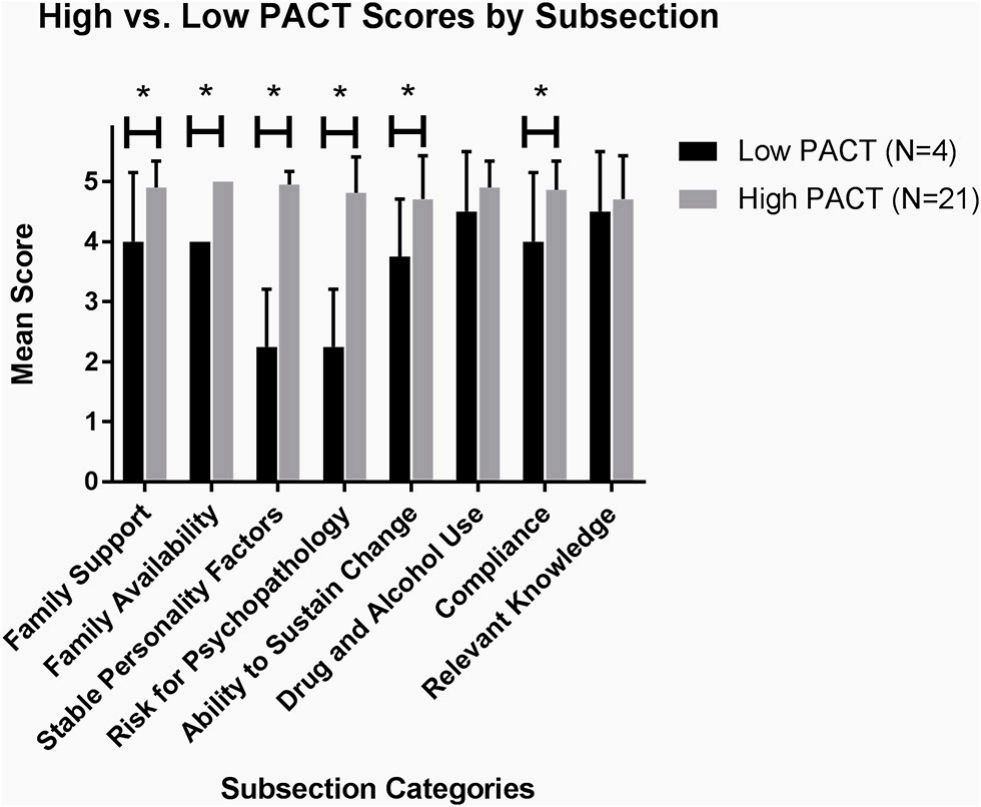
Differences in PACT subcategories between candidates with high vs. low PACT scores. Patients with a low PACT score ≤2 had less family support, family availability, stable personality factors, ability to change and compliance and increased risk for psychopathology than those with high PACT score ≥3. *denotes significance (*p* < 0.05).

When comparing High PACT (≥ 3) vs. Low PACT Scores (≤2), the high PACT mean was 3.52 (SD 0.51) while the low PACT group mean was 1.75 (SD 0.50). The low PACT group had a higher percentage of non-adherence and increased rate of missed appointments per year than the high PACT group (Table 3). Compared to the high PACT group, patients with lower PACT scores had less family support (*p* = 0.01) and family availability (*p* < 0.0010), more unstable personality factors (*p* < 0.001), increased risk for psychopathology (*p* < 0.001), less ability to sustain change (*p* = 0.03), and less medical adherence (*p* = 0.02) (Figure 2). Overall, non-adherence in the low PACT group was associated with less family availability (Odds Ratio 0.08, 95% Confidence Interval 0.01–0.97, *p* = 0.047) and increased risk for psychopathology (OR 0.34, 95% CI 0.13–0.87, *p* = 0.025; Table 4).

**Table 3:** Clinical outcomes between high PACT (≥3) and low PACT scores (≤2) in recipients with a PACT assessment.

	**Low PACT**	**High PACT**	***p***
Post-transplant dialysis (%)	3 (75.0)	6 (28.6)	0.228
Patient death [mean (sd)]	0 (0.0)	0 (0.0)	
Missed appointments per year [mean (sd)]	1.22 (0.68)	0.32 (0.74)	0.062
Non-adherence (%)	3 (75.0)	4 (19.0)	0.094
Graft Failure	0 (0.0)	0 (0.0)	
30-day readmission (%)	0 (0.0)	5 (23.8)	0.682
Hospital stay (days, median [IQR])	14.00 [5.50, 22.25]	7.00 [5.00, 9.00]	0.64
Acute rejection in 3 years of transplant [mean (sd)]	0 (0.0)	4 (19.0)	0.835
Delayed graft function (%)	1 (25.0)	5 (25.0)	0.999

**Table 4:** Odds of non-adherence by PACT subsection in recipients with a PACT assessment.

**Variable**	**Odds ratio**	**95% confidence interval**	***p*-value**
Family support	0.38	0.11–1.41	0.15
Family availability	0.08	0.01–0.97	0.047
Personality factors	0.37	0.13–1.03	0.056
Risk for psychopathology	0.34	0.13–0.87	0.025
Ability to sustain change	1.03	0.34–3.06	0.97
Drug and alcohol abuse	4314	0.00–Inf	0.99
Medical adherence	0.44	0.13–1.53	0.2
Relevant knowledge	1.1	0.32–3.74	0.88

## Discussion

Our study was the first to examine the clinical utility of PACT in a pediatric renal transplantation. Our overall goals were to understand the impact of introducing a standardized psychosocial assessment such as PACT and to elucidate any differences in the clinical outcomes between patients with low and high PACT scores. Similar to adult transplant studies in BMT, lung, and liver recipients, our pilot investigation demonstrated that assessment with PACT was associated with modest improvement in clinical outcomes. Previous studies have shown PACT to be associated with lower in-hospital mortality, shorter length of stay and readmission duration (10, 12, 13, 22, 23). Similar to prior studies, we demonstrated an association between using a standardized assessment, such as PACT, and lower post-transplant morbidity, specifically less acute rejection and non-adherence. Compared to a non-standardized assessment, incorporating PACT into the pre-transplant approach was helpful to identify high risk patients that required more frequent post-transplant monitoring by the medical team including monthly labs, social worker visits, and psychiatry/psychology appointments when appropriate.

The need for pre-transplant psychosocial evaluation has been known for decades, however, standardization of tools or criteria remain insufficient in modern solid organ transplantation (20). Adult measures such as The Stanford Integrated Psychosocial Assessment for Transplant (SIPAT), the Structured Interview for Renal Transplantation (SIRT), and the Transplant Evaluation Rating Scale (TERS) (3–5) have not been studied in pediatrics. PACT has been shown to correlate with outcomes and is among the most studied in adult transplantation. In order to provide one unifying pre-transplant assessment for both pediatric and adults, we evaluated PACT in transplanted children. Our study bridges a gap in the PACT literature and provides important information regarding its potential utility in pediatric renal transplantation to identify those at risk for rejection and non-adherence. Pediatric measures such as the Pediatric Transplant Rating Instrument (P-TRI) and the Psychosocial Assessment Tool (PAT) (24), adapted for pediatric kidney transplantation, suffer from inter-rater variability and did not correlate with clinical outcomes (6, 7, 25).

While outcomes by overall PACT score have been examined, few studies have examined differences in subscales. In bone marrow transplantation (BMT), several subscales were correlated with better outcomes. Foster et al found that adherence was associated with lower in-hospital mortality, shorter length of stay and readmission duration, and faster engraftment. Additionally, higher scores on family availability and on relevant knowledge/receptiveness to education were associated with decreased mortality (12). While our pilot study was not powered for mortality analysis, our results showed that the difference between a high and low PACT score in this pediatric population was driven by family support, innate personality factors, and adherence, similar to what was reported for the BMT population. Additionally, a low PACT score was found in patients with missed appointments and non-adherence. Of those with a PACT score, non-adherence was more likely found in candidates with a higher risk of psychopathology and lack of family support.

Although our pilot study highlights the potential utility of PACT in children, there are limitations. Retrospective analyses are affected by selection and indication biases. By adjusting for HLA mismatch in our analysis, we attempted to minimize significant differences between recipients with no PACT assessment and a PACT assessment. Despite the known association of *de novo* donor specific antibodies (DSA) and non-adherence, baseline DSA and *de novo* DSA were not routinely screened in all patients at our institution until recently (17, 26, 27). Thus, it could not be included as a clinical outcome. Additionally, patients in the two cohorts were transplanted during two different time periods. Patients transplanted between 2005 and 2010 were not assessed with PACT, while those transplanted between 2010 and 2015 were evaluated utilizing PACT. Potential differences in pre- and post-transplant care between these time periods may have impacted our results. One notable difference due to time of transplant was percentage of living related donors in the PACT negative group, most likely due to these recipients undergoing transplant prior to the Share 35 kidney allocation policy. After this policy, which gave pediatrics priority for the best deceased donor allografts, the rate of deceased donor transplantation increased for children (28). We were unable to account for this difference in our final model, but a higher percentage of living donors would be predicted to decrease rates of acute rejection and not increase (29, 30). Lastly, patients without the PACT assessment had longer follow-up time and thus more time to develop poor clinical outcomes. To eliminate this bias, we only evaluated acute rejection episodes within the first 3 years post-transplantation. Although our sample size is small, and our study was not designed to be powered for mortality or graft failure analyses, we found significant findings that warrant further investigation.

In conclusion, renal transplantation requires significant attention to psychosocial factors for successful clinical outcomes. Currently, a standard psychosocial evaluation for transplant candidates across all centers is lacking. Our study highlights that implementation of a standardized measure, such as the PACT, can identify pediatric kidney transplant candidates at risk for poor outcomes, which can align with pre-transplant measures utilized in the adult setting. Thus, future prospective studies may be indicated prior to widespread use.

## Data Availability

The datasets generated for this study are available on request to the corresponding author.

## Author Contributions

KF significantly contributed to the acquisition of data, analysis and interpretation of data for the work. He drafted the manuscript. VC contributed to the acquisition of data and interpretation of data. SN, AC, DW, JF, RG, and DV all contributed to the interpretation of data and critical revisions of the manuscript that significantly added to the scientific content. DC contributed to the analysis and interpretation of data that added to the content of the manuscript. EC contributed to the design of the study, interpretation of data, and drafting of the manuscript. All authors agree to be accountable for all aspects of the work in ensuring that questions related to the accuracy or integrity of any part of the work are appropriately investigated and resolved.

### Conflict of Interest Statement

The authors declare that the research was conducted in the absence of any commercial or financial relationships that could be construed as a potential conflict of interest.
